# The effect of exercise on cytokines: implications for musculoskeletal health: a narrative review

**DOI:** 10.1186/s13102-022-00397-2

**Published:** 2022-01-06

**Authors:** Sophie Docherty, Rachael Harley, Joseph J. McAuley, Lindsay A. N. Crowe, Carles Pedret, Paul D. Kirwan, Stefan Siebert, Neal L. Millar

**Affiliations:** 1 Institute of Infection, Immunity and Inflammation, College of Medicine, Veterinary and Life Sciences, University of Glasgow, 120 University Avenue, Glasgow, G12 8TA Scotland, UK; 2 Sports Medicine and Imaging Department, Clinica Diagonal, C/Sant Mateu 24-26, 08950 Esplugues de Llobregat, Spain; 3 School of Physiotherapy, Royal College of Surgeons in Ireland, Dublin, Ireland; 4 Physiotherapy Department, Connolly Hospital, Dublin, Ireland

**Keywords:** Exercise, Cytokines, Osteoarthritis, Tendinopathy, Inflammation

## Abstract

The physiological effects of physical exercise are ubiquitously reported as beneficial to the cardiovascular and musculoskeletal systems. Exercise is widely promoted by medical professionals to aid both physical and emotional wellbeing; however, mechanisms through which this is achieved are less well understood. Despite numerous beneficial attributes, certain types of exercise can inflict significant significant physiological stress. Several studies document a key relationship between exercise and immune activation. Activation of the innate immune system occurs in response to exercise and it is proposed this is largely mediated by cytokine signalling. Cytokines are typically classified according to their inflammatory properties and evidence has shown that cytokines expressed in response to exercise are diverse and may act to propagate, modulate or mitigate inflammation in musculoskeletal health. The review summarizes the existing literature on the relationship between exercise and the immune system with emphasis on how exercise-induced cytokine expression modulates inflammation and the immune response.

## Summary box

**What’s already known**

- It is widely recognised that regular exercise promotes healthy living in terms of a person’s physical and psychological wellbeing.
- The relationship between exercise and the immune system provides an opportunity to explore the complex interaction between basic physiological and immunological mechanisms in musculoskeletal health and disease.

**What this review shows**

- Activation of the innate immune system occurs in response to exercise and it is proposed this is largely mediated by cytokine signalling.
- Emphasises how exercise-induced cytokine expression modulates inflammation and the immune response.
- Discusses how exercise induced cytokine production is crucial in maintaining musculoskeletal health and how it is altered in disease

## Background

A prima facia case for the benefits of physical exercise is now largely undisputed [1]. It is widely recognised that regular exercise promotes healthy living in terms of a person’s physical and psychological wellbeing; however, the mechanisms by which this is achieved are less well understood. Exercise induces considerable physiological change in the immune system and can be considered an external stress akin to surgery, trauma or sepsis, in the way it induces hormonal and immunological responses, just on a much lesser magnitude [2, 91, 92]. The relationship between exercise and the immune system provides an opportunity to explore the complex interaction between basic physiological and immunological mechanisms in musculoskeletal health and disease.

The immunological changes of exercise can be observed across a range of immune cells and pathways. Exercise has been shown to affect lymphocyte subpopulations, natural killer cell (NK) cell activity, neutrophil functioning and leukocyte trafficking to varying degrees [2]. Importantly, there is a consistent change in the human cytokine profile in response to exercise that is thought to be influential on musculoskeletal health [3].

Cytokines are a diverse family of intracellular signalling molecules that regulate the immune system in both health and disease. A balance between pro-inflammatory and anti-inflammatory cytokines is essential in maintaining tissue homeostasis. Dysregulation of either creates the potential for significant immunopathology [4]. Therefore, cytokine networks must be tightly regulated in order to limit host damage whilst maintaining immunity. In the context of exercise, it is important to consider the impact exercise has on cytokine production and the subsequent effect on the musculoskeletal system. This review will explore the physiological changes in cytokine production induced by exercise with a view to exploring the implications of this in the context of musculoskeletal health and disease.

### Cytokines in acute exercise

The first study suggesting an exercise induced cytokine response was published in 1983 by Cannon and Kluger [5]. In this study, plasma obtained from human subjects following exercise was injected intra-peritoneally into rats resulting in elevated rectal temperature. Samples obtained prior to exercise failed to induce this response. The pyrogenic molecule within the sample was isolated and found to be heat denaturable (suggesting it was likely to be a protein) and 15kDA (consistent with the molecular weight of cytokines). As part of this study, human leukocytes obtained after exercise were incubated in vitro. These leukocytes released a factor into the medium that also elevated body temperature in rats [5]. These results suggested that an endogenous pyrogen was released in response to human exercise—what we now identify as ‘cytokines’.

It is important to note that whilst cytokines are produced throughout the body, in the context of exercise, the primary source of cytokine is the skeletal muscle itself. Skeletal muscle is increasingly recognised as a ‘secretory organ’ and produces cytokines in response to contraction [6, 7]. Over 3000 of these cytokines, termed ‘myokines’, are produced by myocytes and include interleukin 6 (IL-6), IL-7, IL-15 and myostatin, among others [7, 90]. Myokines act primarily in an autocrine and paracrine fashion locally on skeletal muscle, but may also act in an endocrine fashion by communicating with a variety of other tissue types [8]. The finding that skeletal muscle is in fact a secretory organ has generated a new area of research within the exercise field. Researchers have long aimed to find an ‘exercise factor’ which links skeletal muscle contraction with the metabolic changes associated with exercise [9]. The identification of skeletal muscle-derived cytokines could represent the ‘exercise factor’ they were looking for and account for the exercise-induced immune and metabolic changes. The following sections will detail the individual cytokines thought to play a key role in the immunology of acute exercise.

### IL-6

The response of IL-6 to exercise has been studied extensively in the scientific community and considered the pivotal cytokine in exercise physiology [3, 11–13]. The levels of IL-6 increase exponentially (up to 100-fold) in response to exercise and decline rapidly in the period following exercise [3]. The degree of elevation of IL-6 is dependent on multiple factors, such as exercise intensity, duration of exercise, and individuals exercise capacity [13].

#### Role of IL-6 in inflammation and exercise

IL-6 is a pleiotropic cytokine with a broad range of functions in immunoregulation, haematopoiesis and inflammation [14]. It was initially thought to be an important mediator involved solely in the propagation of a pro-inflammatory state. IL-6 mediates pro-inflammatory effects in both the innate and adaptive immune response. IL-6 attracts neutrophils to the site of damage and is involved in B and T-cell differentiation [15]. In addition, IL-6 also inhibits the differentiation of CD4 + /T-cells into T-regulatory cells, thereby limiting the ‘brakes’ on inflammation, helping to propagate the inflammatory state and prevent subsequent resolution. It is involved in the secretion of stress hormones during an inflammatory response, through both acting on the hypothalamus to promote the release of corticotrophin releasing hormone and also acting directly on the adrenal cortex and medulla to release cortisol and catecholamines respectively [93, 94]. Furthermore, IL-6 may also have some role in the secretion of the classic ‘acute phase proteins’ from the liver, including C-reactive protein [94, 95]

Despite its well-characterised pro-inflammatory role, IL-6 exerts an anti-inflammatory effect in the context of exercise [16]. It is proposed that IL-6 mediates its anti-inflammatory effects through the induction of anti-inflammatory cytokines, namely IL-10 and IL-1Ra [10]. From a molecular perspective, it has been suggested that the contrasting actions of IL-6 can be explained through its cellular signalling. It is suggested that its pro-inflammatory effects are mediated by the soluble IL-6 receptor, while its anti-inflammatory effects are mediated by membrane bound receptors, Gp130 [14].

The anti-inflammatory effect of IL-6 can also be seen through its inhibitory effect on TNF-α. It has been long-known and shown experimentally that IL-6 inhibits TNF-α production in vitro in both cultured human monocytes and in monocytic cell lines [17]. This relationship has been verified in IL-6 ‘knockout mice’ and ‘wild type mice’ treated with anti-IL-6, which have markedly elevated circulating levels of TNF-α [18]. This indicates that IL-6 is involved in the regulation of TNF-α levels. In human studies, it was found that the administration IL-6 impairs the TNF-α response. In healthy individuals, when Escherichia coli (E. coli) endotoxin is administered, an elevation of TNF-α is observed. However, IL-6 infusion inhibits the endotoxin-induced TNF-α response. [19] In combination, these studies serve to confirm the effect of IL-6 on TNF-α inhibition, supporting, IL-6 as the primary driver of the anti-inflammatory environment associated with exercise.

#### Secretion and role as a myokine

Prior to the discovery of muscle-derived ‘myokines’, it was proposed that the elevation of systemic IL-6 was a cellular immune response to muscular damage during exercise [10] [20]. As monocytes are the primary source of IL-6 in the immune response to sepsis, monocytes were studied in a laboratory setting to determine their role in the cytokine response to exercise. It was found that the concentration of IL-6 mRNA in monocytes did not elevate following exercises [21], demonstrating that elevation of IL-6 observed in exercise was in fact not derived from traditional circulating immune cells (Table 1).

**Table 1:**
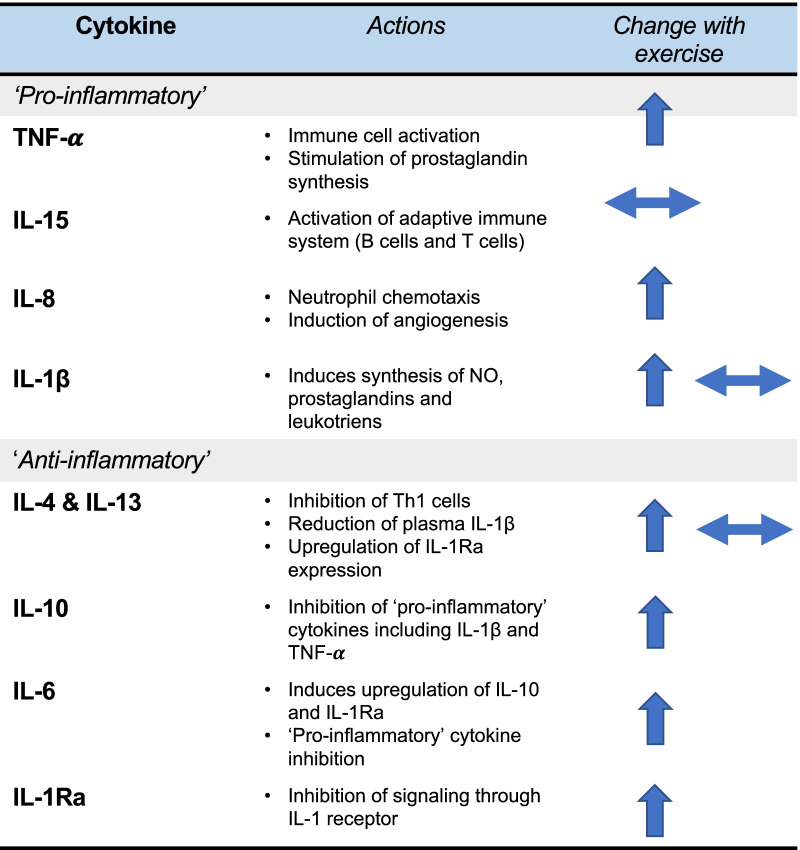
Summary of the roles of key cytokines in relation to exercise

Upwards arrow = upregulated after exercise. Sideways arrow = no change with exercise. More than one arrow indicates conflicting results between studies. Compiled with information from [5, 6, 10, 13, 15–17, 19, 22, 35]

It has subsequently been demonstrated that IL-6 is produced in skeletal muscle itself, as opposed to peripheral immune cells. Exercise activates the transcription of the IL-6 gene in contracting skeletal muscle [6] with IL-6 protein expressed transiently in contracting muscle fibres [22] and released into the circulation from skeletal muscle during exercise [23, 24]. By comparing the plasma concentration of IL-6 in a single exercising leg with the systemic concentration of IL-6, it was found that the concentration of IL-6 was 17-times higher in the exercising leg than in the arterial circulation [3]. This confirmed the status of IL-6 as a myokine in the context of exercise.

Furthermore, the suggestion that the elevation of IL-6 was a response to damage was also refuted. It was found that the difference between IL-6 concentration following both ‘damaging’ (eccentric) and ‘non-damaging’ (concentric) muscular contractions did not differ significantly [10, 25]. This finding, in combination with the fact that exercise does not produce a cellular immune response, demonstrates that the IL-6 response to exercise is a physiological response, rather than a damage phenomenon.

#### IL-6 and glucose metabolism

While IL-6 is primarily involved in creating an anti-inflammatory environment during exercise itself, IL-6 also has distant effects. These effects may explain some of the broad benefits observed with exercise and allow conclusions to be drawn about the role of IL-6 in a wider biological context.

It has been found that IL-6 exerts its action both locally (within the muscle) and when released into the circulation, in a hormone-like fashion in distant organs. In skeletal muscle, IL-6 plays an important role in muscle glucose metabolism during exercise. IL-6 is upregulated in response to low glycogen levels [6]. It has therefore been suggested that IL-6 acts as an ‘energy sensor’ during exercise—upregulating its expression in response to low muscle glycogen [24].

Alongside its local actions, IL-6 acts on the liver and adipose tissue in an endocrine fashion. It has been proposed that IL-6 increases hepatic glucose production during exercise and increase lipolysis in adipose tissues [26]. These distant effects of IL-6 are important in the maintenance of homeostatic glucose concentrations in the face of increased glucose uptake by skeletal muscle [27]. These findings can still be replicated when other known inducers of hepatic gluconeogenesis are accounted for [28].

In addition to its role in glucose metabolism, IL-6 is also involved in the generation of alternative energy sources during exercise. IL-6 acts to increase AMP-activated protein kinase (AMPK) activity in skeletal muscle [29]. The AMPK pathway stimulates fatty acid oxidation and increases glucose uptake by skeletal muscle cells [30]. IL-6 is also involved in the enhancement of glucose transporter type 4 (GLUT4) expression. GLUT4 is an inducible glucose receptor and provides an alternative energy source for muscle when glycogen sources are scarce. Together, these pathways highlight the essential role the upregulation of IL-6 plays in skeletal muscle metabolism during exercise [30].

It is important to note that these metabolism studies focus on the action of IL-6 during muscular contraction during exercise. In contrast, infusions of recombinant IL-6 in humans at rest fail to induce any changes in glucose metabolism, implying that an additional, unidentified factor is needed for IL-6 to influence glucose metabolism [31]. Recently, it has been shown that exercise-induced visceral fat loss in obese people is inhibited by IL-6 receptor blockade with tocilizumab, indicating this process is mediated dependent on IL-6 signalling [32]. These studies demonstrate the complexity of the role of IL-6 in exercise and suggests an area for further research in fully establishing its role in muscle and general metabolism.

**Fig. 1:**
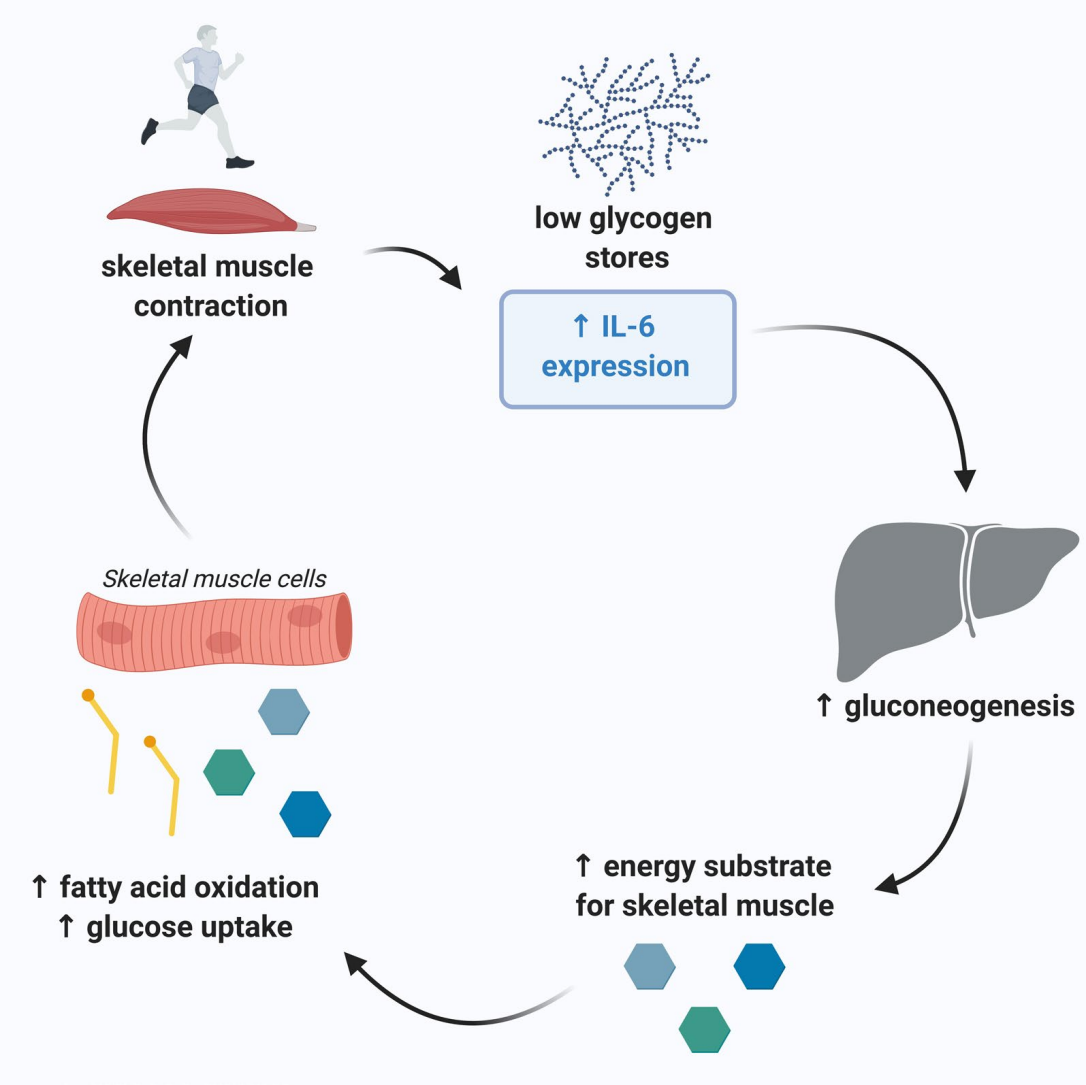
Illustration of the actions of exercise-derived IL-6 on local and systemic metabolism

A diagrammatic representation of the role of the IL-6 in muscle metabolism is shown in Fig. 1.

These anti-inflammatory and metabolic effects of IL-6 may provide a mechanism to help explain the well documented beneficial effects of exercise in health and why exercise reduces the susceptibility to, or improves the symptoms of, inflammatory conditions [33].

### TNF-α and IL-1β

TNF-α and IL-1β have both been studied in the context of exercise with a view to understanding the immunological environment of exercise. Both TNF-α and IL-1β are considered to be classic pro-inflammatory cytokines and are released in response to cellular damage. Through their action in vivo, they ultimately stimulate a pro-inflammatory response through the activation of immune cells and increasing systemic prostaglandins [34].

A rise in the concentration of TNF-α and IL-1β is not seen in moderate exercise, although they have been shown to increase in prolonged or strenuous exercise [35]. After a marathon race, the concentration of TNF-α and IL-1β was shown to increase twofold. By comparison, the concentration of anti-inflammatory IL-6 increased 50-fold [36]. It can therefore be concluded that whilst strenuous exercise induces an increase in the pro-inflammatory cytokines, TNF-α and IL-1β, it is largely counteracted by the induction of anti-inflammatory cytokines leading to an overall anti-inflammatory response.

### IL-10 and IL-1Ra

Evidence has shown that circulating levels of both interleukin 10 (IL-10) and IL-1Ra both rise in the period following exercise [10] and their release is likely upregulated in response to IL-6 [91, 96, 97]. IL-10 and IL-1Ra play a role in immune regulation and have also been implicated in contributing to the anti-inflammatory response to exercise.

IL-10 is considered a classic anti-inflammatory cytokine. It is proposed that IL-10 suppresses cytokines through both direct inhibition of the action of pro-inflammatory cytokines, as well as by preventing cytokine synthesis [36]. Using post-translational mechanisms, IL-10 is able to block nuclear factor kappa-B (NF-κB), a transcription factor termed the ‘master regulator’ of the immune system. In doing so, IL-10 prevents the generation of pro-inflammatory cytokines [37]. IL-10 inhibits a number of cytokines, including TNF-α and IL-1β, which is an important consideration in the context of exercise given that the levels of these cytokines are low despite elevated IL-6 [38].

In contrast to the action of IL-10, which influences a spectrum of cytokines, IL-1Ra mediates its effects on IL-1 alone. IL-1Ra inhibits signal transduction by competitively binding to IL-1 receptor complex [39], thus preventing IL-1 binding and mediating its pro-inflammatory effects.

### IL-4

IL-4 is an anti-inflammatory cytokine that may contribute to the overall anti-inflammatory environment observed in exercise. IL-4 mediates its action primarily through the inhibition of Th1 cells, reduction of plasma IL-1**β** and upregulation of IL-1Ra [40].

The IL-4 response to exercise is less well characterised than the key mediators involved in exercise. Studies have shown no change in IL-4 expression in the immediate aftermath of exercise [40]. However, it has been suggested that IL-4 may be involved in long-term muscular adaptations to exercise [41]. Through regular training, the expression of IL-4 within muscles has been found to increase over time following repeated individual exercise sessions [42]. This suggests that through regular training, muscles become more able to mediate some of their anti-inflammatory profile through the upregulation of IL-4.

### IL-13

IL-13 has been studied alongside IL-4 due to the similarity in the actions of both cytokines. Like IL-4, IL-13 also inhibits T helper type 1 (Th1) cells, reduces plasma IL-1b and upregulates IL-1Ra [40]. Alongside its anti-inflammatory role, it has been suggested that IL-13 has a distinct role in exercise and metabolism. Knudsen et al. report that endurance training in mice increases the local production of IL-13 within muscles, which, through the activation of downstream pathways, leads to improved muscle fatty acid utilisation and mitochondrial biogenesis [43]. This response was not observed in mice which lacked IL-13. The potential anti-inflammatory and metabolic roles of IL-13 in exercise may provide an important focus for understanding the metabolic conditioning that can be observed through regular exercise. Further studies are required to investigate the relationship between exercise and IL-13 expression in humans.

### IL-8

IL-8 belongs to the CXC family of chemokines and is primarily involved in neutrophil migration, as well as angiogenesis in vivo [44]*.* In the context of exercise, IL-8 is produced locally within the muscle during exercise, with a minimal systemic IL-8 response only observed following intense exercise with an eccentric component owing to the associated pro-inflammatory response in this setting [12]. The role of IL-8 in angiogenesis is distinct from its pro-inflammatory actions [45]. IL-8 acts via the stimulation of CXC receptors 1 and 2(CXCR1 and CXCR2). CXRC2 is expressed by microvascular endothelial cells and is responsible for IL-8 induced angiogenesis [46]. It has been shown that exercise induces CXCR2 mRNA and protein expression in the vascular endothelial cells of muscles [47]. This suggests muscle-derived IL-8 exerts its action locally, primarily to stimulate exercise-induced angiogenesis through CXCR2.

### IL-15

IL-15 is a cytokine which acts as both an immunoregulatory mediator and as a growth factor. IL-15 is highly expressed in skeletal muscle following exercise [48] and has been shown to act in an anabolic fashion by increasing the production of myosin within skeletal muscles [49]. This response has been shown to be up-regulated by strength training [12]. Interestingly, IL-15 has also been shown to play a role in the reduction of adipose tissue mass − a direct juxtaposition to its anabolic functions [50]. It has therefore been proposed that IL-15 acts to regulate the muscle to fat interactions which ultimately modulates the effects of exercise on the ratio of fat to lean body composition [51].

### Overall cytokine profile of acute exercise

**Fig. 2:**
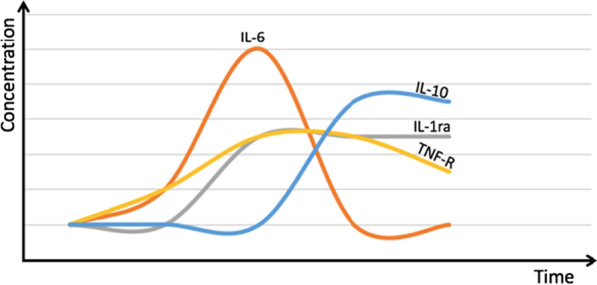
Illustration of circulating cytokines released in response to exercise

In addition to the levels of cytokines, the dynamic sequence following acute exercise also needs to be considered. The overall sequence of cytokine release in response to exercise involves an initial rise in the plasma concentration of IL-6, followed by a subsequent rise in the concentration of IL-1Ra, IL-10 and soluble TNF-receptors (TNF-R) (Fig. 2). This sequence of pro-inflammatory cytokines followed by release of anti-inflammatory cytokines is also seen in sepsis and acute inflammatory conditions but in contrast to sepsis, there is no preceding or accompanying increase in TNF-α in moderate acute exercise.

### Chronic changes due to exercise

The acute changes in cytokine production during exercise are fairly well characterised, although there is some variation based on the intensity and type of exercise. However, less data is available on the long-term effects of regular exercise on the cytokine profile in humans. The reasons for this include the difficulty in determining the extent to which the cytokine production is a consequence of physical fitness per se, associated lifestyle factors or a direct consequence of an exercise regime. Furthermore, determining a true ‘resting’ cytokine profile creates logistical issues when recruiting subjects willing to abstain from exercise for the duration of a study [2].

The existing studies indicate that the cytokine profile of an individual changes with chronic exercise, although the degree to which they change remains widely debated.

With regards to changes in pro-inflammatory cytokines, the ATTICA study found that regular physical activity reduces basal plasma IL-6 and TNF-α in an urban population [52]. One proposed mechanism for the cytokine changes observed is that regular exercise leads to a reduction in body fat. Adipocytes are a major source of pro-inflammatory cytokines, including TNF-α and IL-6 [53], although this reduction also occurs in the absence of weight loss or changes in body composition, suggesting other mechanisms, including potentially also direct anti-inflammatory effects on immune cells, are likely to be involved [54].

Alongside the observed reduction in pro-inflammatory cytokines, regular exercise has been shown to increase the circulating concentration of anti-inflammatory cytokines. Prokopchuk et al. demonstrated that IL-1Ra, IL-4 and IL-13 were significantly increased with high-intensity training over a 6-week period [41], with the increase in IL-1Ra replicated by Forti et al. [55].

These findings support a relationship between low physical activity and inflammation, in which low levels of physical activity are associated with chronic low-grade inflammation, which may contribute to the increased cardiovascular risk associated with sedentary lifestyles [52, 56–59]. This relationship between regular exercise, cytokine profiles and inflammation is one which, with further research, may provide further insight into the long-term benefits of exercise, particularly in regards to inflammatory, cardiovascular and other chronic diseases, and deliver additional opportunities to intervene in these conditions.

### The effect of excessive exercise

As mentioned, there are a multitude of undeniable benefits that regular, moderate exercise can provide, extending from metabolic to cardiac to psychological. However, exercise is not without its dangers as well, especially when performed to an excessive level. Excluding injury, there are several levels of severity to the harm exercise can cause. ‘Overreaching’ is when there is a temporary drop in performance as a result of excessive training, which recovers after a period of rest [98]. Overtraining syndrome (OTS) is more severe, when the deterioration in performance persists despite adequate rest [94, 98]. This is also associated with an array of other symptoms, including but not limited to: increased susceptibility to injury, fatigue, sleep disruption, weight loss, muscle tenderness, weakness, depression, anxiety, difficulty concentrating and loss of appetite. In addition to this, there are biochemical and immunological changes which occur, with many reportedly experiencing an increased susceptibility to infection and illness [94, 98, 101]. There are many proposed mechanisms for this, but thus far, no overarching explanation for the phenomenon seems to have been discovered.

In 2000, Smith introduced the ‘Cytokine Hypothesis of Overtraining’, arguing that the collection of symptoms and biochemical/immunological changes experienced with OTS are caused by pro-inflammatory cytokines, mainly IL-1β, IL-6 and TNF-α [94]. It has been reported extensively that excessive exercise leads to an increase in pro-inflammatory cytokines [91, 92, 94, 98–103]. Excessive exercise, particularly with the use of eccentric contractions (often demonstrated using methods such as downhill running) have been shown to increase pro-inflammatory cytokines in the serum, within the muscle tissue itself, and within articular cartilage [102, 103]. It is plausible, and in fact likely that these cytokines are responsible for many of the symptoms experienced in OTS, such as low mood, loss of appetite, elevated cortisol levels etc. [94]. However, studies have demonstrated that within 2 weeks of OTS diagnosis, pro-inflammatory cytokine levels normalised with rest whilst diminished performance levels remained, suggesting other processes are also at work [104].

Many other mechanisms have been shown to contribute to varying degrees, including muscle glycogen depletion, free radical damage to contractile proteins, reduced mitochondrial capacity and so on [94, 98, 101]. Exercise is known to cause physiological damage to muscle, termed ‘microtrauma’, which allows the muscle to repair and remodel to adapt to increased loads, resulting in hypertrophy and angiogenesis [94, 98, 99, 109, 111]. Tightly regulated inflammation is the process which enables this and involves myeloid cell invasion of the damaged muscle [109–111]. Macrophages are the main component of this adaptive response, and cytokines thought to play a role in this include TNF-α and IFNγ, which cause a pro-inflammatory macrophage response, and IL-4, IL-10 and IL-13, which cause an anti-inflammatory macrophage response and actually inhibit optimal repair [113]. When adequate recovery is not allowed, chronic inflammation can set in, characterised by elevated intramuscular levels of TNF-α, IFNγ, IL-6 and IL-10, with muscle damage persisting for weeks [98, 109]. Chronically elevated IL-6 is known to downregulate expression of proteins involved in the mitochondrial electron transfer chain as well as upregulate oxidative ability of neutrophils, releasing more reactive oxygen species (ROS) causing free-radical damage to the contractile protein filaments within myofibrils, thus impeding muscle function [98, 112].

Intimately interlinked with the cytokines are the white blood cells. Nieman introduced the J-shaped curve in 1994, suggesting that moderate exercise reduces the chance of infection whilst excessive exercise increases the risk above that of a sedentary individual [114]. The infection in question is an upper respiratory tract infection (URTI), since this is the most common infection experienced by athletes in both summer and winter sports [106]. Exercise in moderate amounts is known to be immunoprotective [97]. In response to an acute bout of strenuous exercise (e.g. running a marathon), many immune cell changes are witnessed. A biphasic neutrophilia occurs, firstly due to demargination then cortisol-induced neutrophil release from bone marrow; initial neutrophil degranulation and increased oxidative burst activity proceeded by a decrease below baseline in both, most likely mediated by IL-6 surges; NK cell surge likely mediated by catecholamine-induced demargination; decreased nasal and salivary IgA levels likely due to increased sympathetic activity; decreased MHC-II expression and toll-like receptor expression on monocytes/macrophages; increased pro-inflammatory and anti-inflammatory cytokines including TNF-α, IL-6, IL-1β, IL-4, IL-10, IL-1ra etc.; upregulation of T_H_2 lymphocytes and concurrent downregulation of T_H_1 mediated by the pattern of raised cytokines [94, 97, 99–101, 103, 107]. These changes may last anywhere from 2-24 h or beyond and are often proportional in magnitude and duration to the intensity and length of the exercise [99]. It is often argued that the changes seen increase susceptibility to infection and thus ratifies the ‘open window’ [99, 105, 106]. It is hypothesised that these changes, when compounded during an intense period of training without adequate rest, lead to overall immunosuppression and hence create the J-shape curve where high exercise levels lead to increased infections [106].

Several controversies still remain unsolved regarding this issue, as the clinical significance of the initial immune changes remain contested. Some papers argue that a single bout of excessive exercise leads to increased URTI risk, such as Nieman who found increased URTI rates in those who participated in the Los Angeles Marathon, with the risk 2-times higher in those who trained over 97 km per week in the lead up to the race [107]. It should be pointed out though that only 1 in 7 experienced any symptoms, so the vast majority did not [107]. This may be related to the findings that high exercise loads, in addition to causing inflammation, also increase circulating concentrations of the anti-inflammatory cytokines IL-4 and IL-10, which are known to cause immunosuppression and correlate with increased URTI incidence [101, 116–118]. Others, however, argue that the symptoms are in fact reactivation of a virus not completely cleared before running the race, or even non-infective inflammation as a result of increased exposure to irritants and pollutants whilst exercising [99, 106, 108]. In most studies, none of the infections were clinically diagnosed or verified using viral swabs, relying solely on the self-reporting and self-diagnosis of participants [97]. Other factors, such as pathogen exposure, psychological stress, sleep hygiene and diet were not taken into account, which are also known to have an effect on immune function [97, 106].

Whilst it is generally accepted that OTS has an associated infection risk, with these infections arriving more frequently and lasting longer than healthy individuals, the J-shape curve theory weakens when elite athletes are taken into consideration [105, 108, 115, 119]. This makes sense, as Mårtensson et al. point out that in order to maintain a competitive training schedule of 500–800 h per year, athletes cannot afford to be stricken with illness very often [108]. When extrapolating this data out, the curve becomes more of an S-shape, suggesting that the high level of activity associated with immunosuppression be more applicable to recreational and sub-elite athletes rather than professional, elite athletes [105, 108, 115, 119].

### Exercise Induced cytokines and the musculoskeletal system

As described, exercise-related cytokines are largely derived from within the musculoskeletal system therefore have a role to play in musculoskeletal health and its pathology. Alongside this, many musculoskeletal conditions are characterised by local or systemic inflammation which, in turn, creates a complex relationship between the pre-existing immune environment and the additional immune modulation derived from exercise.

### Osteoarthritis

Osteoarthritis (OA) is a disease of the joint in which excessive ‘wear and tear’ results in increased degradation of articular cartilage and the underlying subchondral bone. In terms of pathophysiology, OA is the result of a disruption to the homeostatic process of synthesis and degradation of articular cartilage, extracellular matrix and subchondral bone. OA commonly affects the knees, hips and small joints of the hands [60] and can result in significant pain and morbidity for patients. It is estimated that 8.5million patients in the UK suffer from OA, with incidence increasing as related conditions such as obesity also increase in prevalence [61]. Treatment options include analgesia and physiotherapy in the earlier stages, although a significant number of patients will progress to requiring total joint replacement to improve their quality of life [62].

While osteoarthritis is not traditionally considered an inflammatory disease, the role of local cytokines in the pathophysiology of the condition has become increasingly recognised in recent years. Studies have found evidence of elevated IL-1 family members, TNF-α and IL-6 in the synovium, subchondral bone and cartilage of patients with OA, suggesting a role for inflammation in this pathology [63, 64]. These cytokines are released by cells in adipose tissue and act as part of the system to negatively regulate cartilage synthesis. IL-1 and IL-6 inhibit collagen II synthesis while increasing the activity of matrix-metalloprotinases (MMPs) – collagen digesting enzymes. The discovery of the role of IL-6 in cartilage regulation has prompted research into the effects of IL-6 blockade in OA [63]. A phase 3 clinical trial has recently been completed which examined the efficacy of tocilizumab, an IL-6 receptor inhibitor, in the treatment of OA [65]. The results from this trial are yet to be published, but they may provide further insight into the extent IL-6 influences OA pathology.

With relation to exercise, there has been extensive research into how exercise influences outcomes in OA. Meta-analyses have reported that exercise improves pain, function and quality of life for patients with OA [66] and in the UK the National Institute for Health and Care Excellence (NICE) guidelines recommend exercise therapy as part of first line treatment [62].

Despite these recommendations, little research is available to indicate how exercise improves OA at the molecular level. Data from the ADAPT trial showed that higher levels of inflammatory markers, including IL-6, are associated with poorer patient-reported outcomes, independent of body mass index [64]. However, a causal link has not been proven. Further research is warranted to determine the role of exercise-induced cytokines in the pathophysiology and treatment of OA, which may provide an evidence base for the best use of exercise in the management of OA.

### Rheumatoid arthritis

A similar relationship can be seen in rheumatoid arthritis (RA), the most prevalent form of inflammatory arthritis. RA is a systemic auto-immune condition that primarily affects the synovial joints. RA is characterised by synovitis, autoantibody production, cartilage and bone destruction in addition to systemic inflammation and features such as cardiovascular and pulmonary manifestations. [67]

Cytokines play a central role in the pathogenesis of RA. Amongst others, TNF-α and IL-6 have been implicated as key drivers of the systemic inflammation observed in RA patients. It has been proposed that the dysregulated, persistent production of IL-6 contributes to the production of autoantibodies, local inflammation and systemic effects [68].

In contrast to its role in exercise, IL-6 is thought to be largely pro-inflammatory in RA. Inhibition of IL-6 using anti-IL-6 receptor antibody (tocilizumab) is an effective and widely used first-line biological therapy in the treatment of active moderate-to-severe RA [69].

Given that reducing systemic IL-6 is beneficial in RA, it would be reasonable to hypothesise that exercise, and its associated rise in IL-6, may potentially worsen the symptoms of RA. However, multiple studies have shown that exercise programmes can reduce symptoms of pain and stiffness in patients with RA. [70] Furthermore, exercise has benefits in terms of improving functional ability and psychological well-being. Crucially there is no evidence to suggest that exercise exacerbates disease activity [71]. These findings indicate that exercise, both resistance and aerobic, should be included in the treatment of RA. This is reflected in clinical guidelines which recommend that patients with RA should participate in regular exercise [72] [73].

The question therefore remains, how does exercise—which is known to induce IL-6 production and release—result in benefits for patients with RA? The answer may come from the transient nature of IL-6 release in exercise and lack of accompanying increase in TNF-α [74]. Unlike in active RA where IL-6 is chronically elevated, IL-6 rises throughout the period of exercise, before rapid removal from the circulation in the post-exercise period (Fig. 2) [75]. During its short period of activity, IL-6 induces the upregulation of anti-inflammatory cytokines, including IL-1Ra, which persist in the circulation and induce a longer lasting anti-inflammatory effect [31]. The overall net anti-inflammatory effect of exercise may account for some of the symptomatic improvement for patients with RA, despite an accompanying increase in IL-6.

### Psoriatic arthritis

Exercise has also been recognised as beneficial for symptomatic management in patients with Psoriatic Arthritis (PsA). PsA is a chronic inflammatory joint disease, affecting joints, tendons and ligaments in some patients with skin psoriasis [76]. Whilst PsA can present similarly to RA, PsA represents a distinct immunopathology with a cytokine profile that is distinct from RA [77].

While the IL-23/IL-17 pathway is now recognised as being key in psoriatic disease [78], L-6 is also raised and implicated in PsA. IL-6 is elevated in the synovium of patients with PsA and has a role in promoting T-helper cell differentiation, thus propagating the inflammatory cycle. [79] However, clazakizumab, an IL-6 inhibitor, failed to demonstrate a dose response in a phase II study. [80]

The failure of IL-6 blockade suggests that, although IL-6 is involved, it is not central to the initiation of the inflammatory response [81]. It has been proposed that the inhibition of IL-6 leads to the overproduction of other pro-inflammatory cytokines, such as IL-17, locally within the skin and joints further driving pathology [82].

It is unclear whether the benefit of exercise in PsA is also due to the net anti-inflammatory effect proposed for RA or if low levels of IL-6 may help stabilise some of the other pro-inflammatory cytokines in affected tissues.

### Tendinopathy

Tendinopathy is a common musculoskeletal presentation in both the general practice and orthopaedic setting. The condition can cause significant pain on movement and loss of function for patients, as well as weakening of the tendon itself [83]. This results in a predisposition to tears which may require surgical intervention. Current treatment for tendinopathy is centred around tendon loading programs to restore the capacity of the affected tendon; however, while this has been shown to be of benefit, for many patients loading programs fail to achieve adequate improvements, with the result many patients continue to suffer from chronic pain and dysfunction.

The role of inflammatory cytokines in tendinopathy has been widely debated; however, it is increasingly recognised as playing a significant role in the early stages of tendon disease [84]. As tendinopathy is most commonly a result of overuse, it often affects people who exercise regularly. It is therefore of interest to determine how exercise-induced cytokines influence tendinopathy.

As in skeletal muscle, exercise induces IL-6 expression in tendon tissue. It has been shown that following exercise, IL-6 is upregulated in healthy tendon but not in tendinopathic tendon [85]. Additionally, mice lacking the IL-6 gene were found to have inferior tendon healing compared to wild-type mice [86]. This provides evidence for the role of exercise-induced cytokines in normal tendon physiology and adaptation to exercise, and suggests a role for IL-6 in tendon healing and failed response to loading stimuli.

Studies have shown that the use of eccentric loading exercises improve outcomes in patients with tendinopathy [87]. Recently, meta-analysis has found strong and consistent evidence indicating eccentric loading exercises are the most effective treatment for tendinopathies [88]. Additionally, it was found that eccentric exercise increases the cross-sectional area and stiffness of tendon [89]. Tendon tissue has been shown to upregulate IL-6 and TGF-β in response to mechanical stimulation, with TGF-β proposed as the driving force behind the structural changes observed with repeated eccentric exercise. TGF-β has been shown to induce remodelling in a variety of tissues by promoting epithelial-mesenchymal transformation, suggesting this may also be the case in tendon [89]. Further research is required to understand the physiological role of IL-6 and TGF-β in tendons in order to help determine the point at which exercise begins to promote an inflammatory environment.

The apparently paradoxical roles of IL-6 in chronic musculoskeletal conditions, such as tendinopathy, OA and inflammatory arthritis, highlights the diversity of the musculoskeletal system’s response to cytokines which regulate it, both in response to exercise and in overall health. Currently exercise is mainly used for symptom control and functional improvement in these conditions; an improved understanding of the relationship between exercise-related immune changes and musculoskeletal health may facilitate evidence-based therapeutic exercise strategies targeting the inflammatory pathology of these conditions, in conjunction with pharmaceutical agents.

## Conclusions

Exercise induces significant physiological changes in the immune system, including characteristic cytokine responses. Most notable is a marked elevation in muscle-derived IL-6 which, despite being traditionally regarded as a potent pro-inflammatory cytokine, helps orchestrate an anti-inflammatory immune response in exercise.

Despite IL-6 and pro-inflammatory cytokines being implicated in various chronic musculoskeletal conditions, this exercise-induced increase in IL-6 does not appear to lead to inflammatory exacerbations in these conditions, with exercise generally conferring beneficial effects. This interaction raises intriguing questions about how to best utilize this effect for the treatment of these conditions and offers exciting research opportunities within the fields of sports medicine and immunobiology, both clinically and experimentally. With continued research, exercise and its associated cytokine profile may provide an effective therapeutic avenue that will lessen the burden of musculoskeletal disease.

## Data Availability

N.L.M has access to all the data and data are available upon request.

## Abbreviations

NK: Natural killer (cell)
kDA: Kilodalton
IL: Interleukin
IL-1Ra: Interleukin-1 receptor antagonist
Gp130: Glycoprotein 130
TNF: Tumour necrosis factor
AMPK: AMP-activated protein kinase
GLUT: Glucose transporter
T_H_1: T-helper 1 cell
T_H_2: T-helper 2 cell
mRNA: Messenger RNA
OTS: Overtraining syndrome
IFN: Interferon
ROS: Reactive oxygen species
URTI: Upper respiratory tract infection
IgA: Immunoglobulin A
MHC: Major histocompatibility complex
OA: Osteoarthritis
MMP: Matrix-metalloproteinase
NICE: National Institute for Clinical Excellence
RA: Rheumatoid arthritis
PsA: Psoriatic arthritis
